# Baseline markers of cortical excitation and inhibition predict response to theta burst stimulation treatment for youth depression

**DOI:** 10.1038/s41598-023-45107-1

**Published:** 2023-11-04

**Authors:** Prabhjot Dhami, Sylvain Moreno, Paul E. Croarkin, Daniel M. Blumberger, Zafiris J. Daskalakis, Faranak Farzan

**Affiliations:** 1 School of Mechatronic Systems Engineering, Simon Fraser University, 250-13450 102 Avenue, Surrey, BC V3T 0A3 Canada; 2 Temerty Centre for Therapeutic Brain Intervention, Centre for Addiction and Mental Health, 1001 Queen St. W, Toronto, ON M6J 1A8 Canada; 3 Institute of Medical Science, Faculty of Medicine, Medical Sciences Building, University of Toronto, 1 King’s College Circle, Toronto, ON M5S 1A8 Canada; 4 School of Interactive Arts and Technology, Simon Fraser University, 250-13450 102 Avenue, Surrey, BC V3T 0A3 Canada; 5 Circle Innovation, 1200-555 W. Hastings Street, Vancouver, BC V6B 4N6 Canada; 6 College of Medicine and Science, Mayo Clinic, Rochester, MN 55905 USA; 7 Department of Psychiatry, University of Toronto, 250 College Street, 8th Floor, Toronto, ON M5T 1R8 Canada; 8 Department of Psychiatry, University of California San Diego, 9500 Gilman Dr, La Jolla, CA 92093 USA

**Keywords:** Predictive markers, Depression

## Abstract

Theta burst stimulation (TBS), a specific form of repetitive transcranial magnetic stimulation (TMS), is a promising treatment for youth with Major Depressive Disorder (MDD) who do not respond to conventional therapies. However, given the variable response to TBS, a greater understanding of how baseline features relate to clinical response is needed to identify which patients are most likely to benefit from this treatment. In the current study, we sought to determine if baseline neurophysiology, specifically cortical excitation and/or inhibition, is associated with antidepressant response to TBS. In two independent open-label clinical trials, youth (aged 16–24 years old) with MDD underwent bilateral dorsolateral prefrontal cortex (DLPFC) TBS treatment. Clinical trial one and two consisted of 10 and 20 daily sessions of bilateral DLPFC TBS, respectively. At baseline, single-pulse TMS combined with electroencephalography was used to assess the neurophysiology of 4 cortical sites: bilateral DLPFC and inferior parietal lobule. Measures of cortical excitation and inhibition were indexed by TMS-evoked potentials (i.e., P30, N45, P60, N100, and P200). Depression severity was measured before, during and after treatment completion using the Hamilton Rating Scale for Depression—17. In both clinical trials, the baseline left DLPFC N45 and P60, which are believed to reflect inhibitory and excitatory mechanisms respectively, were predictors of clinical response. Specifically, greater (i.e., more negative) N45 and smaller P60 baseline values were associated with greater treatment response to TBS. Accordingly, cortical excitation and inhibition circuitry of the left DLPFC may have value as a TBS treatment response biomarker for youth with MDD.

Clinical trial 1 registration number: NCT02472470 (June 15, 2015).

Clinical trial 2 registration number: NCT03708172 (October 17, 2018).

## Introduction

Major depressive disorder (MDD) has a lifetime prevalence of approximately 11.0% in adolescents and youth (15–24 years old)^1^. It has been estimated that up to 50% of such patients do not respond to conventional first-line treatments, including medication, psychotherapy, or a combination of both^2^. For such individuals, repetitive transcranial magnetic stimulation (rTMS), a non-invasive form of brain stimulation, may be a treatment option^3^. Recent work suggests that a relatively novel form of rTMS, called theta burst stimulation (TBS)^4^, is a safe, feasible, and clinically effective treatment for MDD in youth^5,6^. However, the response rate to TBS remains variable, and determining what type of patient is most likely to respond to this treatment is an ongoing and active area of research^7^. Given its potential as an alternative treatment option for youth with MDD, a greater understanding of what baseline features may influence clinical response to TBS is needed.

From a neurophysiological perspective, cortical inhibition, which refers to inhibitory interneurons attenuating the activity of other neurons^8^, is thought to influence the effects of TBS^9^. Depending on the orientation and strength of the TMS-induced current flow, TMS can depolarize inhibitory interneurons in the cortex^10^. In the context of animal studies, TBS has been found to modulate the expression of proteins found in inhibitory interneurons^11,12^. Additional evidence includes TBS being reported to disinhibit sensory responses and reducing interneuron-related protein expression^13^. Accordingly, the literature suggests that the effects of TBS may in part be related to the cortical inhibition properties of the targeted site.

To probe the inhibitory and excitatory circuitries of the human brain, concurrent transcranial magnetic stimulation and electroencephalography (TMS-EEG) may be used. Following a single TMS pulse, TMS-evoked potentials (TEPs) are elicited, with the canonical TEPs being the P30, N45, P60, N100, and P200. Pharmacological TMS-EEG studies suggest the N45 and N100 to reflect GABAergic neurotransmission^14–19^. Other TEPs are believed to reflect excitatory mechanisms, such as the P60, which has been linked to glutamatergic neurotransmission^19^. Given that GABAergic neurotransmission is believed to be critical with regards to the effects of TBS^20^, TMS-EEG is a tool well suited to search for related treatment response biomarkers.

In the current study, we sought to investigate potential neurophysiological-based TBS treatment response biomarkers in youth with MDD. Data were collected and assessed independently from two clinical trials: one in which youth with MDD underwent 10 bilateral dorsolateral prefrontal cortex (DLPFC) TBS treatment sessions, and another in which youth with MDD underwent 20 bilateral DLPFC TBS combined with cognitive exercise treatment sessions. Prior to beginning treatment, all participants underwent TMS-EEG testing. The baseline TMS-EEG session included single pulse stimulation of four cortical sites: bilateral DLPFC and inferior parietal lobules (IPL). The IPLs were chosen as control sites as they were not the sites of stimulation but are still implicated in MDD due to belonging to the default mode network^21^. As magnetic-based brain stimulation treatments, including TBS, are believed to exert their therapeutic effect in part by acting upon inhibitory mechanisms^11,22–24^, we hypothesized that TEPs that reflect similar mechanisms (i.e., N45 and N100) from the left and right DLPFC would be associated with treatment response. Specifically, we hypothesized that greater baseline cortical inhibition (as reflected by a greater negativity of the N45 and N100) at the sites of stimulation (i.e., bilateral DLPFC) would be associated with greater treatment response.

## Methods

### Clinical intervention

#### Clinical trial design

The first clinical trial, titled “Efficacy and Biological Targets of Response to rTMS Therapy in Youth Depression”, was designed as an open label 2-week TBS treatment trial and was registered (https://clinicaltrials.gov/ct2/show/NCT02472470). The second clinical trial, titled “rTMS and Cognitive Training in Youth MDD”, was registered (https://clinicaltrials.gov/ct2/show/study/NCT03708172) and designed as an open label 4-week TBS treatment with engagement in a cognitive exercise.

For the first clinical trial (Supplementary Fig. 1), 20 youth MDD patients were recruited to undergo an open label 2-week TBS treatment, which consisted of 10 bilateral DLPFC TBS sessions; treatment sessions were conducted once a day, 5 days a week, over the course of the 2 weeks^5^. For this trial, as it was the first to investigate the application of TBS for treatment of MDD in youth, a shorter treatment duration was used in part to establish its safety and feasibility. The second clinical trial (Supplementary Fig. 2) was designed as an open label 4-week TBS treatment with engagement in a cognitive exercise. The treatment consisted of 20 bilateral DLPFC TBS followed by engagement in cognitive exercise sessions (i.e., once daily, 5 days a week, over the course of 4 weeks)^6,24^. Thirty youth MDD patients were randomized to receive active or sham cognitive exercise. Following the completion of each TBS session, participants completed the cognitive exercise component. Cognitive exercise took place after each treatment session, within 30 min of completing TBS. The active cognitive exercising treatment arm consisted of a computerized program, which combined motor, perceptual, and cognitive tasks. The program consisted of five components: rhythm exercises, voice awareness, music theory, musical ear training, and singing exercises. Over the course of a single session, the components became progressively more difficult. The sham cognitive exercise component consisted of lessons from Khan Academy, specifically of Grade 10 mathematics. Patients engaged in the cognitive exercises on an iPad following their TBS treatment session.

For both clinical trials, daily TBS treatment sessions involved 1800 intermittent TBS (iTBS) pulses applied to the left DLPFC and 1800 continuous TBS (cTBS) pulses applied to the right DLPFC. Conventional TBS parameters were used; specifically, TBS consisted of a 3-pulse 50-Hz burst applied every 200 ms (i.e., 5 Hz), with iTBS consisting of a 2 s train of bursts repeated every 10 s, and cTBS consisting of an uninterrupted train of bursts^4,25^. Coordinates used (in Talairach) were *x* =  − 35, *y* = 45, and *z* = 35 for the left DLPFC, and *x* = 34, *y* = 46, and *z* = 35 for the right DLPFC. Stimulation was conducted at 80% of each participant’s active motor threshold. The administration order of whether iTBS or cTBS was applied first with was randomized for each participant^25^.

#### Sample

Across both clinical trials, all eligible participants were outpatients on stable treatment (medication or psychotherapy) for at least 4-weeks prior to testing; between the ages of 16–24; had a Mini-International Neuropsychiatric Interview (MINI) confirmed diagnosis of single or recurrent MDD; a Hamilton Rating Scale for Depression (HRSD) − 17 score of 18 or greater; at least one failed antidepressant trial in the current episode as defined by the Antidepressant Treatment History Form; and were safe to receive TMS according to the TMS adult safety screening questionnaire. Exclusion criteria included a lifetime MINI diagnosis of autism spectrum disorder, bipolar I or II, current psychotic symptoms, delusional disorder, obsessive compulsive disorder, schizophrenia, or schizoaffective disorder; a history of epilepsy or any other major neurological disorder; a history of MINI-determined substance use disorder within the last 3 months; concomitant major unstable medical illness; acute suicidality on assessment; or concomitant medications considered to be study confounds including benzodiazepines, mood stabilizers, and stimulants. All participants provided written informed consent and the protocols were approved by the Centre for Addiction and Mental Health Research Ethics Board (REB) and in accordance with the Declaration of Helsinki.

### TMS-EEG testing

#### Design

All participants underwent TMS-EEG testing prior to beginning their respective treatment. Each participant’s anatomical MRI images were used to guide TMS coil positioning during TMS-EEG testing using neuro-navigation (Brainsight TMS Navigation; Rogue Resolutions).

#### Stimulation sites

Participants were stimulated with 80 monophasic TMS pulses at 4 cortical sites independently: the bilateral DLPFC and bilateral IPL. The TMS inter trial interval was 5 s with no jitter. Stimulation sites for each cortical region was determined using the following MNI coordinates (left DLPFC: − 35, 45, 38; right DLPFC: 35, 45, 38; left IPL: − 52, − 54, 36; right IPL: 52, − 51, 43). TMS pulses were administered by a 7-cm figure-of-8 coil through two Magstim 200 stimulators (Magstim Company Ltd). As each site was stimulated independently, the order of site testing was randomized for each participant for their respective TMS-EEG test session. The intensity of stimulation was set at the determined threshold required to elicit 1 mV motor evoked potentials (MEPs).

#### Intensity

The stimulation intensity applied at each of the four cortical sites was determined by identifying the intensity required to evoke 1 mV MEPs. First, the optimal coil location and orientation was identified to elicit MEPs. This was measured and determined using electromyography from the abductor pollics brevis in accordance with previously published methods and standards in the field^26^. The resting motor threshold (rMT) was determined for the left and right hemisphere independently and was defined as the stimulator output necessary to evoke an MEP of more than 50 μV in at least five out of ten trials^27^. The intensity required to evoke 1 mV MEP in 15 consecutive trials was estimated at 120% of the rMT. If this value failed to yield an average of 1 mV MEP over 15 trials, the stimulator output was adjusted until the 1 mV was determined. The adjustment procedure consisted of increasing or decreasing the stimulator output and obtaining the 15-trial average again (with the goal of achieving a value range between 0.5 and 1.5 mV). If the desired range was not achieved, the intensity was adjusted again, and another 15-trial were collected and averaged. This process was repeated until an appropriate stimulator output was determined.

#### Controlling for TMS-EEG-related artifacts

For all stimulation conditions, a thin layer of foam was placed over the TMS coil in order to minimize electrode movement and auditory TMS-EEG artifact induced by bone conduction^28^. Participants also wore earplugs during TMS-EEG testing to reduce the auditory-evoked potential artifact associated with the TMS discharge^28–30^.

### EEG recording and preprocessing

EEG was collected using a 64-channel Synamps 2 EEG system. All electrodes were referenced to an electrode positioned posterior to the Cz electrode. Before beginning TMS-EEG testing, the impedance for all electrodes was lowered to $\le 5 \mathrm{k}$ Ω. EEG data was then recorded in DC mode with a sampling rate of 20 kHz. EEG data was preprocessed using the EEGLAB^31^ and TMSEEG^32^ toolboxes as implemented in MATLAB.

In the first step, the recorded signal was down sampled to 1000 Hz. It was then epoched into separate trials which consisted of EEG data between − 1000 ms before and 1000 ms after the TMS pulse. Each trial was baseline corrected using the mean between − 1000 ms and − 110 ms before the TMS pulse. This period was chosen for baseline correction as the data was free of noise and artifacts created by the TMS pulse. Data from − 5 ms before the TMS pulse to 10 ms after the pulse, the period which contains the TMS stimulation artifact, were then removed. In the first round of cleaning, channels and trials that were deemed as being contaminated (e.g., high amplitude noise) were removed. Independent component analysis was then conducted in order to remove TMS-related decay artifacts. The selection of components was done so in accordance with established criteria in the field of TMS-EEG signal processing^32,33^. The data was then filtered using a notch filter (with a band-stop of 55–65 Hz) and a bandpass filter (1–80 Hz) to remove 60 Hz line noise and slow and high-frequency artifacts, respectively. A second round of independent component analysis was then conducted to further remove recurring noise-related components such as eye blinks, eye movements, muscle activity, electrode discontinuity, or electrocardiography. A second round of removing bad channels and trials was also conducted. Finally, channels that were removed were interpolated, and all channels were average referenced.

### Statistical analysis

For our statistical analysis, to assess how baseline neurophysiology was potentially related to treatment response over time, a linear mixed-effects modelling approach was used^34^. In each clinical trial, for each TMS-EEG stimulation paradigm, a linear mixed-effects model was fit for each TEP at each electrode. In each linear mixed-effects model, age, sex, TEP amplitude, time (i.e., HRSD-17 scores across all clinical assessment points for that trial), and an interaction between TEP amplitude and time were included as fixed effects; subjects were included as a random effect. For quantification of each TEP’s (i.e., P30, N45, P60, N100, and P200) magnitude, the mean amplitude was taken across the following time windows, respectively: 25–35 ms, 35–50 ms, 50–70 ms, 70–130 ms, and 150–250 ms, as based on previous literature^35,36^. The outcome variable consisted of HRSD-17 scores, which was the primary clinical outcome measure of interest, at all assessment time points for each trial. Specifically, for clinical trial one, which consisted of 10 TBS sessions, HRSD-17 scores at baseline, after treatment session 5, and post-treatment (i.e., after treatment session 10) were included in the outcome variable. For clinical trial two, which consisted of 20 TBS sessions, HRSD-17 scores at baseline, after treatment session 5, after treatment session 15, and post-treatment (i.e., after treatment session 20) were included in the outcome variable.

Given we were interested in how baseline TEPs may relate to clinical response to TBS over time, the interaction term between TEP and time was of primary interest. For each stimulation paradigm, 300 p-values related to this interaction term were generated (5 TEPs across 60 electrodes). Accordingly, for each stimulation paradigm, the results were corrected for multiple comparisons using the false discovery rate (FDR) procedure^37^. For an interaction term to be considered significant, it had to have had an FDR-corrected p-value of less than 0.05. Additionally, to further reduce potential spurious significant interaction terms, and given the spatial smoothing of EEG signals, for each TEP, significant models were clustered together based on their spatial (i.e., electrode) adjacency. Only clusters with a size of equal to or greater than 2 are reported and interpretated. Given the design differences between the two clinical trials, all the analyses were conducted for each clinical trial separately. All statistical analyses were performed using MATLAB 2021b (Mathworks Ltd, USA).

## Results

### Demographic and clinical characteristics

Demographic and clinical data for patients from clinical trial one and two are presented in Table 1**.** For the first clinical trial, 17 of the initial 20 participants completed the treatment. For the second clinical trial, 26 of the initial 30 participants completed the treatment. The number of participants with available baseline TMS-EEG data varied between the four stimulation sites: left DLPFC (clinical trial one n = 20; clinical trial two n = 25), right DLPFC (clinical trial one n = 20; clinical trial two n = 25), left IPL (clinical trial one n = 14; clinical trial two n = 26), and right IPL (clinical trial one n = 14; clinical trial two n = 24). This was due to two reasons: baseline TMS-EEG data was lost for one participant from the second clinical trial, and the remaining participants found certain stimulation paradigms intolerable and thus testing was stopped. Clinically, both groups exhibited a significant decrease in HRSD-17 scores following their respective treatment (clinical trial one t-value = 7.68 and p-value < 0.001; clinical trial two t-value = 13.84 and p-value < 0.001).

**Table 1:** Baseline demographic and clinical information of youth MDD participants from clinical trials one and two.

Characteristic	Clinical trial one (10 TBS sessions)	Clinical trial two (20 TBS sessions)
Sample size, no	20	30
Age, mean (SD)	20.95 (2.59)	20.40 (2.69)
Number of males, no	10	11
Baseline HRSD-17, mean (SD)	22.35 (2.93)	21.60 (1.98)
HRSD-17 following treatment 5, mean (SD)	17.10 (5.73)	15.44 (2.87)
HRSD-17 following treatment 10, mean (SD)	13.53 (5.00)	NA
HRSD-17 following treatment 15, mean (SD)	NA	12.35 (4.32)
HRSD-17 following treatment 20, mean (SD)	NA	8.77 (4.20)

### Baseline TEPs as predictors of response to TBS

In both datasets, significant interactions between TEPs and time were only found for stimulation of the left DLPFC; no significant interaction terms were found for the stimulation paradigms of right DLPFC, left IPL, and right IPL. The TEP waveforms, distribution of TEP amplitude values, and TEP-related topoplots for all four stimulation paradigms and for each clinical trial can be found in the Supplementary Materials (Supplementary Figs. 3–8).

**Figure 1:**
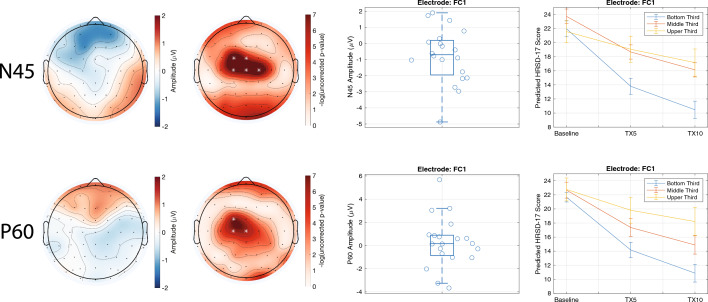
TEPs from Stimulation of the Left DLPFC and Their Association with Treatment Response to TBS in Clinical Trial One. Each row illustrates the relationship between the left DLPFC N45 (top row) and P60 (bottom row) and treatment response to TBS. The left topoplot shows the mean amplitude values. The right topoplot shows the p-value of the interaction term of interest (i.e., TEP × time) as obtained from the linear mixed effects model. Electrodes illustrated with a white asterisk are those that were significant at a FDR-corrected p-value of less than 0.05. Boxplots illustrate the distribution of the amplitude values across all participants for an exemplar electrode. Line plots depict the significant interaction from the exemplar electrode by splitting participants into groups of three based on their TEP amplitude and illustrating their predicted treatment response over time. Error bars represent one standard error of the mean.

For clinical trial one, significant interaction terms of interest were found for the N45 and P60 TEPs (Fig. 1). To delineate the nature of the significant interaction terms in the linear mixed-effects models, patients were split into three groups based on TEP values for exemplar electrodes. This allowed us to gain insight into the interaction as to how the magnitude of baseline TEPs were related to response to TBS over time. With regards to the N45, although the three baseline N45-defined groups had similar baseline HRSD-17 scores, the bottom third group, which had the greatest (i.e., most negative) N45, had a better treatment response over time (fixed effect parameters for this model from an exemplar electrode are presented in Supplementary Table 1). With regards to the P60, a similar trend emerged. Specifically, smaller P60 values at baseline were associated with better treatment response over time (fixed effect parameters for this model from an exemplar electrode are presented in Supplementary Table 2).

**Figure 2:**
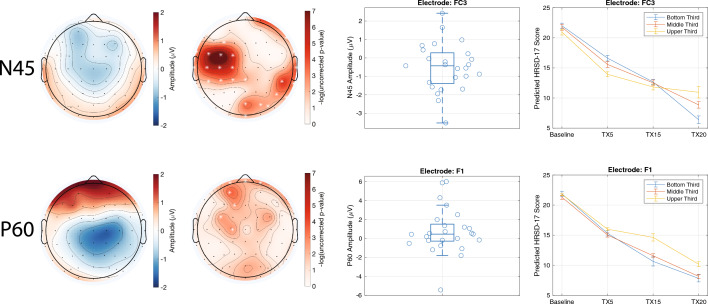
TEPs from Stimulation of the Left DLPFC and Their Association with Treatment Response to TBS in Clinical Trial Two. Each row illustrates the relationship between the left DLPFC N45 (top row) and P60 (bottom row) and treatment response to TBS. The left topoplot shows the mean amplitude values. The right topoplot shows the p-value of the interaction term of interest (i.e., TEP × time) as obtained from the linear mixed effects model. Electrodes illustrated with a white asterisk are those that were significant at a FDR-corrected p-value of less than 0.05. Boxplots illustrate the distribution of the amplitude values across all participants for an exemplar electrode. Line plots depict the significant interaction from the exemplar electrode by splitting participants into groups of three based on their TEP amplitude and illustrating their predicted treatment response over time. Error bars represent one standard error of the mean.

For clinical trial two, significant electrodes were found across all five TEPs (i.e., P30, N45, P60, N100, P200). Due to their statistical significance across both datasets, we focus on the N45 and P60 (Fig. 2). Once again, to delineate the nature of the significant interaction terms in the linear mixed-effects models, patients were split into three groups based on TEP values from exemplar electrodes^35^. With regards to the N45, the group with the highest (i.e., most negative) baseline N45 ultimately had a better treatment response following the clinical trial (fixed effect parameters for this model from an exemplar electrode are presented in Supplementary Table 3). For the P60, a smaller baseline P60 was associated with better treatment response over time (fixed effect parameters for this model from an exemplar electrode are presented in Supplementary Table 4).

## Discussion

The prevalence of MDD in youth has significantly increased in recent years^38^. Given the limitations of conventional therapies^2^, alternative treatments options with improved efficacy are needed for the youth MDD population. TBS is a promising treatment option, but clinical response remains variable. In the context of youth MDD, due to the substantial time needed for remission to be achieved^39^ and the heightened risk for suicidal behavior in this patient population^40^, it is critical to develop antidepressant treatment response biomarkers to reduce time spent on ineffective treatments^41^. Accordingly, a greater understanding of what baseline features are linked to clinical response to TBS are needed. In the current study, we searched for potential neurophysiological-based treatment response biomarkers for bilateral DLPFC TBS in youth with MDD. Our findings suggest that baseline cortical excitation and/or inhibition, as measured by TEPs, may be related to the clinical response following treatment with bilateral DLPFC TBS.

Our hypothesis was that the magnitude of cortical inhibition related TEPs of the left and right DLPFC would be linked to treatment response. This was based on previous studies which found associations between the left DLPFC N45 and N100, TEPs linked to cortical inhibition^14–19^, with treatment response to magnetic-based brain stimulation treatments^22,23^. Indeed, theoretical models on the mechanism of TBS propose a critical role of GABAergic interneurons in both the facilitatory and inhibitory effects of TBS^20^. This hypothesis was partially supported, as across both trials, the left DLPFC N45 was found to be a significant predictor of bilateral DLPFC TBS treatment response. TMS-EEG pharmacological studies have found the N45 TEP to be linked to GABA_A_ neurotransmission^14^ as well as the inhibitory and excitatory balance between GABAergic and glutamatergic neurotransmission^14,15,19^. Accordingly, our finding suggests that the integrity of the GABAergic system in the DLPFC may influence the therapeutic effects of TBS. This is in line with evidence that suggests the therapeutic mechanisms of magnetic stimulations may in part be related to transsynaptic interneuron functioning^42,43^. Thus, in the context of magnetic-based brain stimulation treatments, including TBS, a more robust interneuronal network may allow for better transsynaptic activation of neuronal circuits, which may result in a greater treatment response.

Additional evidence that the effects of TBS may in part rely on inhibitory interneuron functioning comes from both animal and human studies. For example, TBS has been found to increase the expression of enzymes related to GABA synthesis (e.g., GAD65 and GAD67), which are expressed in cortical inhibitory interneurons^12^. When applied to the human motor cortex, cTBS has been reported to acutely increase local cortical GABA concentrations^44^. iTBS of the prefrontal cortex was also found to increase the N100 TEP^45–47^, which is believed to reflect GABA_B_ neurotransmission^14^. Thus, the literature seems to suggest that TBS influences inhibitory interneuron functioning. However, we must note that in our study, only baseline left DLPFC TEPs were significantly related to treatment response; no TEPs following stimulation of the right DLPFC were found to be related to treatment response. This may be due to the left DLPFC receiving iTBS, and the right DLPFC receiving cTBS. Indeed, it is likely that the neurophysiological therapeutic mechanisms of iTBS for treatment of MDD differs from that of cTBS^20,48^. However, we cannot rule the additional factor of the region stimulated; it may be that the neurophysiology of the left DLPFC is critical in predicting treatment response, whereas the right DLPFC is to a lesser degree.

Our initial hypothesis was that the level of cortical inhibition at sites of stimulation would predict treatment response. However, the P60 (as elicited by stimulation of the left DLPFC) was also found to be a significant predictor of response across both clinical trials. The P60 was recently found to be modulated by anti-glutamatergic drugs; specifically, Perampanel was found to reduce the amplitude of the P60, suggesting the P60 to reflect excitatory neurotransmission^19^. Although we did not initially hypothesize excitatory mechanisms to predict treatment response to TBS, this finding still aligns with our speculation that greater baseline cortical inhibition is associated with greater treatment response to TBS. This is because a smaller, and not greater, P60 was linked to greater treatment response across both clinical trials. Accordingly, given that TEPs are believed to reflect a balance between both excitatory and inhibitory neurotransmission, greater cortical inhibition, which we speculated would predict treatment response, may be reflected not only by a greater (i.e., more negative) N45, but also by a smaller P60.

There are certain limitations to the current study. Firstly, there was no sham control treatment arm, nor treatment arms with only iTBS or cTBS, leaving us unable to infer whether the baseline left DLPFC N45 and P60 are predictors of response specific to bilateral DLPFC TBS. There is also the possibility of other reasons which may have led to a decrease in clinical symptoms over time, including, but not limited to, placebo-expectation, regression to the mean, and natural variation of illness course. An additional potential limitation is the contamination of TEPs by somatosensory and auditory artifacts^30^. However, these confounds may not be associated with our primary findings given that a growing body of evidence suggests that only later TEPs (i.e., N100 and P200) are partially contaminated by sensory and auditory artifacts^28,30,49,50^. The early latency of the N45 and P60 likely precludes them from such contamination. We also note that the two clinical trials had study design differences, thus making it difficult for our results to be purely interpreted in a discovery and validation framework. Furthermore, another limitation may be regarding the extent of information available in the registered trials. Our registered trials only specified our aims, including to identify biological targets and predictors of response to TBS. However, the hypothesis that TEPs related to cortical inhibition will be related to TBS response was not provided in the registrations. We also note that the sample sizes between the four sites probed with TMS-EEG varied such that there were more data for the DLPFC than the IPL, particularly for clinical trial one. Finally, the overall sample size within each clinical trial was limited, thus calling for replication of our findings with larger sample sizes in future studies.

TBS has potential as a novel treatment for MDD in youth, but there is also a need for the development of TBS treatment response biomarkers to help identify which patients are likely to benefit from such treatment. Our findings suggest that baseline cortical excitation and inhibition at the site of TBS application may influence clinical response, and thus offer a potential treatment response biomarker for TBS in the context of treating MDD in youth.

### Supplementary Information

Supplementary Information.

## Data Availability

The data that support the findings of this study are available from the corresponding author upon reasonable request.
