# Supplementary material for: Baseline markers of cortical excitation and inhibition predict response to theta burst stimulation treatment for youth depression
Source: Sci Rep. 2023 Nov 4;13:19115. doi: 10.1038/s41598-023-45107-1 (PMC10625527; doi:10.1038/s41598-023-45107-1)
Supplement: Supplementary file 1 — Supplementary Information. [file 41598_2023_45107_MOESM1_ESM.pdf]

Supplementary Materials:

Baseline Markers of Cortical Excitation and Inhibition Predict Response to Theta Burst Stimulation Treatment for Youth Depression

Prabhjot Dhami, PhD[1,2,3], Sylvain Moreno, PhD[4,5], Paul E. Croarkin, DO, MS[6], Daniel M. Blumberger, MD, MSc[2,3,7], Zafiris J. Daskalakis, MD, PhD[2,3,7,8] & Faranak Farzan, PhD[1,2,3,7*]

1. School of Mechatronic Systems Engineering, Simon Fraser University, 250-13450 102 Avenue, Surrey, British Columbia, V3T 0A3, Canada.
2. Temerty Centre for Therapeutic Brain Intervention, Centre for Addiction and Mental Health, 1001 Queen St. W, Toronto, Ontario, M6J 1A8, Canada.
3. Institute of Medical Science, Faculty of Medicine, University of Toronto, Medical Sciences Building, 1 King's College Circle, Toronto, Ontario, M5S 1A8, Canada.
4. School of Interactive Arts and Technology, Simon Fraser University, 250-13450 102 Avenue, Surrey, British Columbia, V3T 0A3, Canada.
5. Circle Innovation, 1200-555 W. Hastings Street, Vancouver, British Columbia, V6B 4N6, Canada.
6. Mayo Clinic, College of Medicine and Science, Rochester, MN, 55905, USA.
7. Department of Psychiatry, University of Toronto, 250 College Street, 8th floor, Toronto, Ontario, M5T 1R8, Canada.
8. Department of Psychiatry, University of California San Diego, 9500 Gilman Dr, La Jolla, CA, 92093, USA.

***Corresponding Author**: Faranak Farzan, PhD, P.Eng., School of Mechatronic Systems Engineering, Simon Fraser University, 250-13450 102 Avenue, Surrey, British Columbia, V3T 0A3, Canada, 1-778-782-7571, (faranak.farzan@sfu.ca)

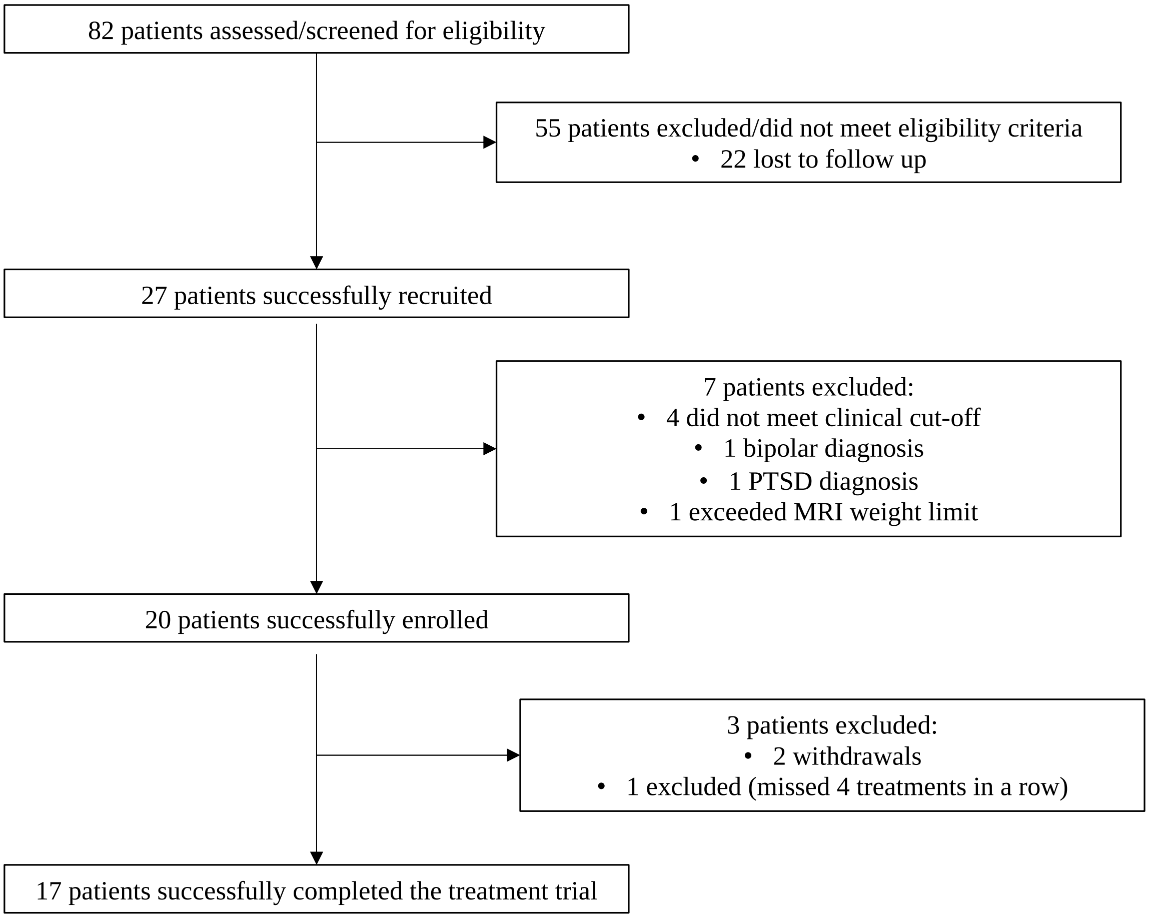

Supplementary Figure 1. Trial Profile for Clinical Trial One.

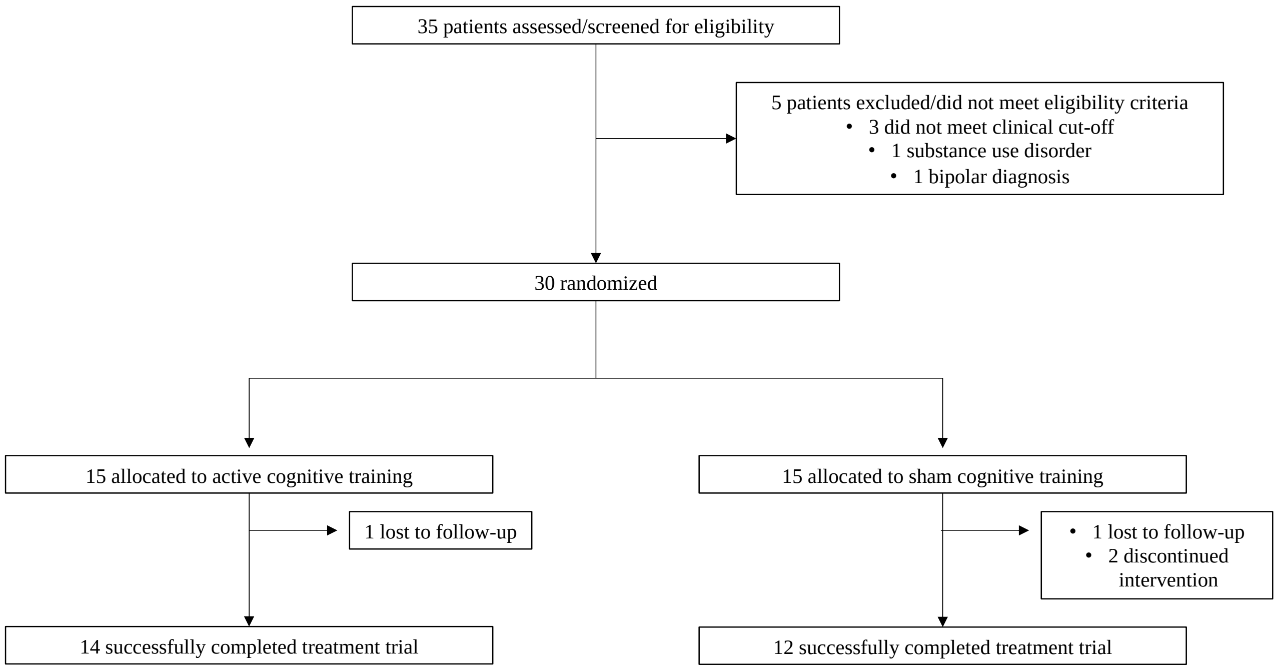

Supplementary Figure 2. Trial Profile for Clinical Trial Two.

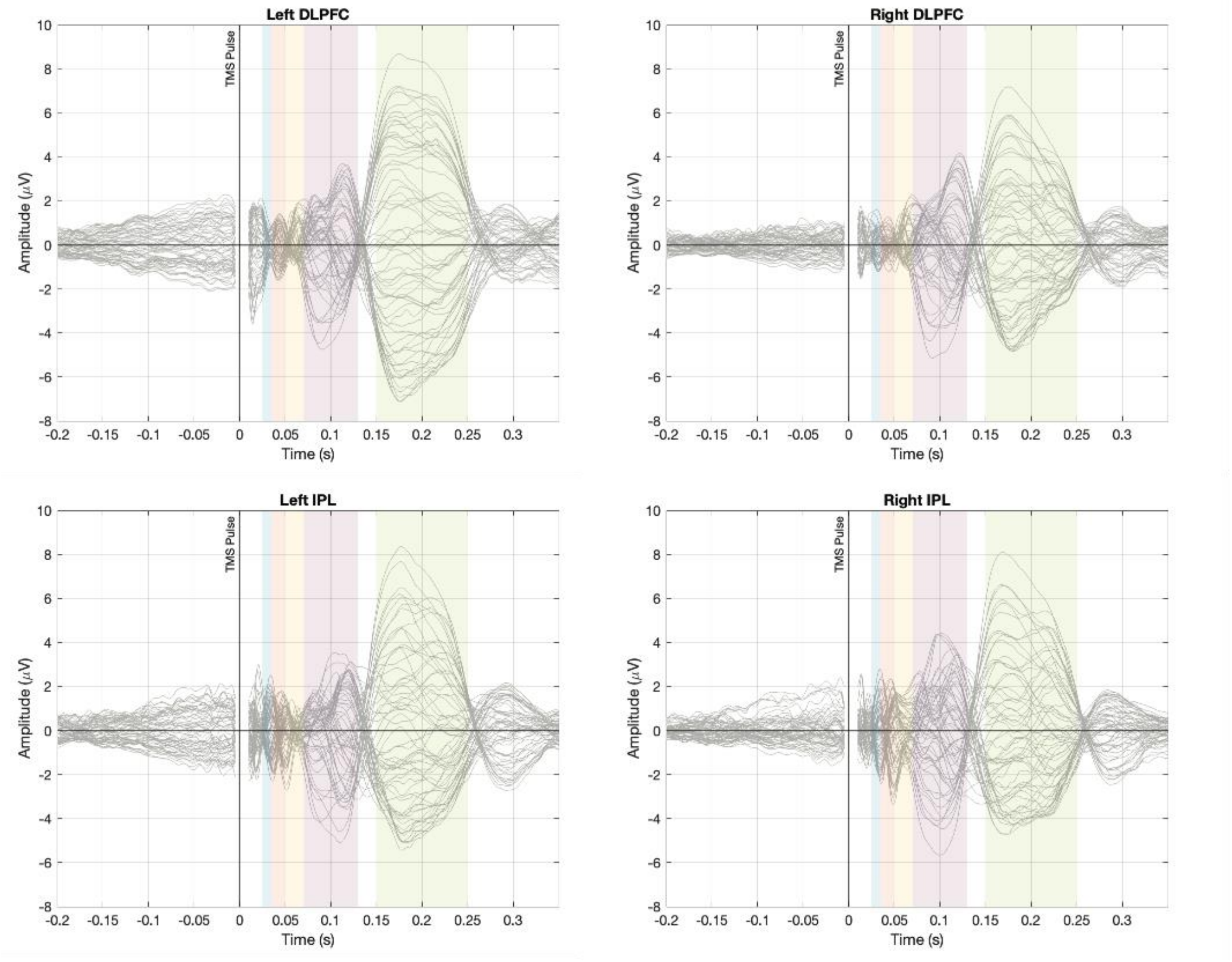

Supplementary Figure 3. TEP Plots Following Stimulation of the Four Cortical Sites in Clinical Trial One. Butterfly plots illustrate the TEPs (averaged across all trials for each participant and then averaged across all participants) from all four cortical sites of interest, as obtained from clinical trial one. Each line represents an electrode. Different coloured shading represents the time windows used for calculating the mean amplitude for the P30, N45, P60, N100, and P200 TEPs.

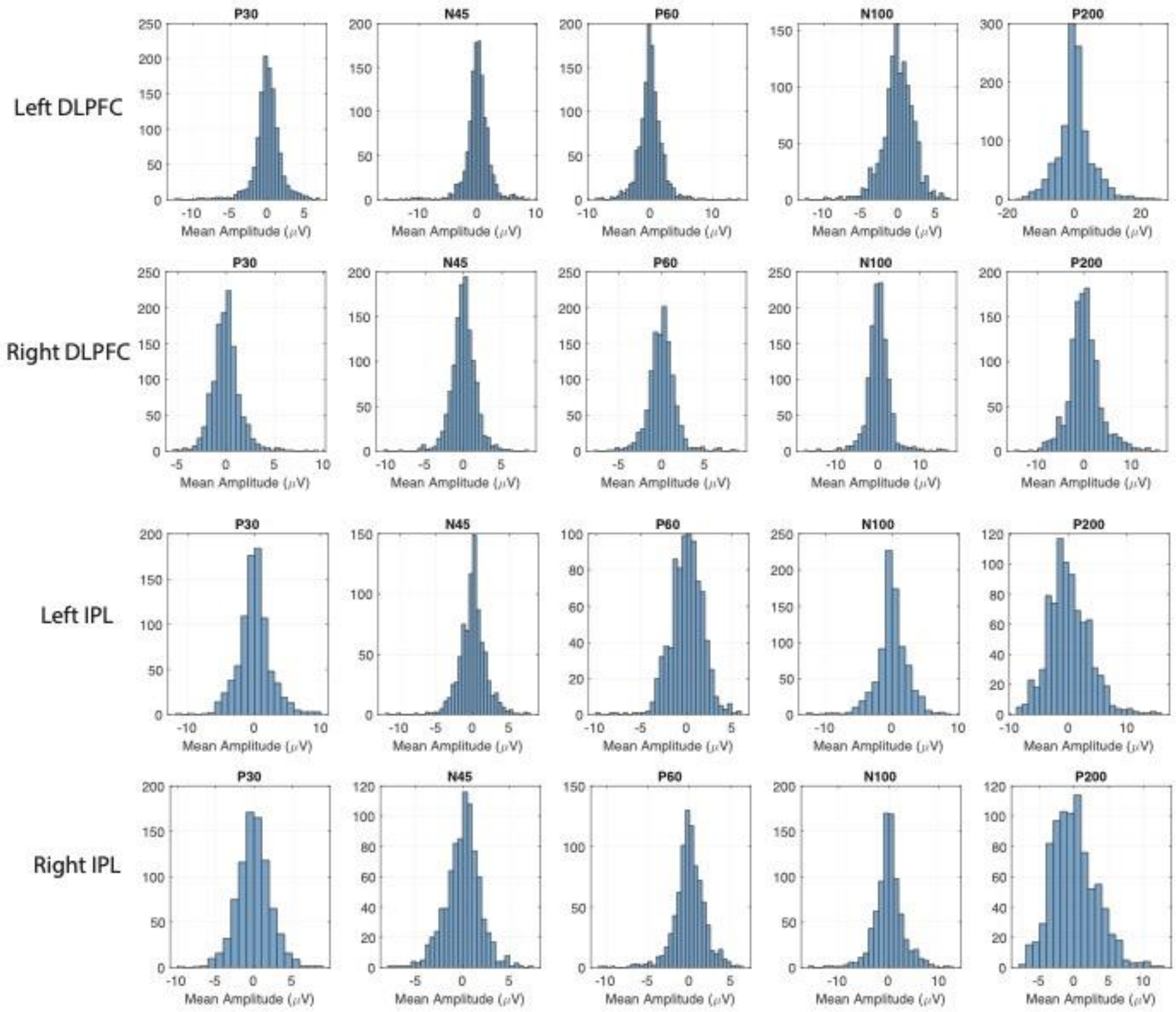

Supplementary Figure 4. Histogram Plots of all TEP Amplitude Values Following Stimulation of the Four Cortical Sites in Clinical Trial One. Each histogram plot includes the TEP amplitude value from each electrode for each participant.

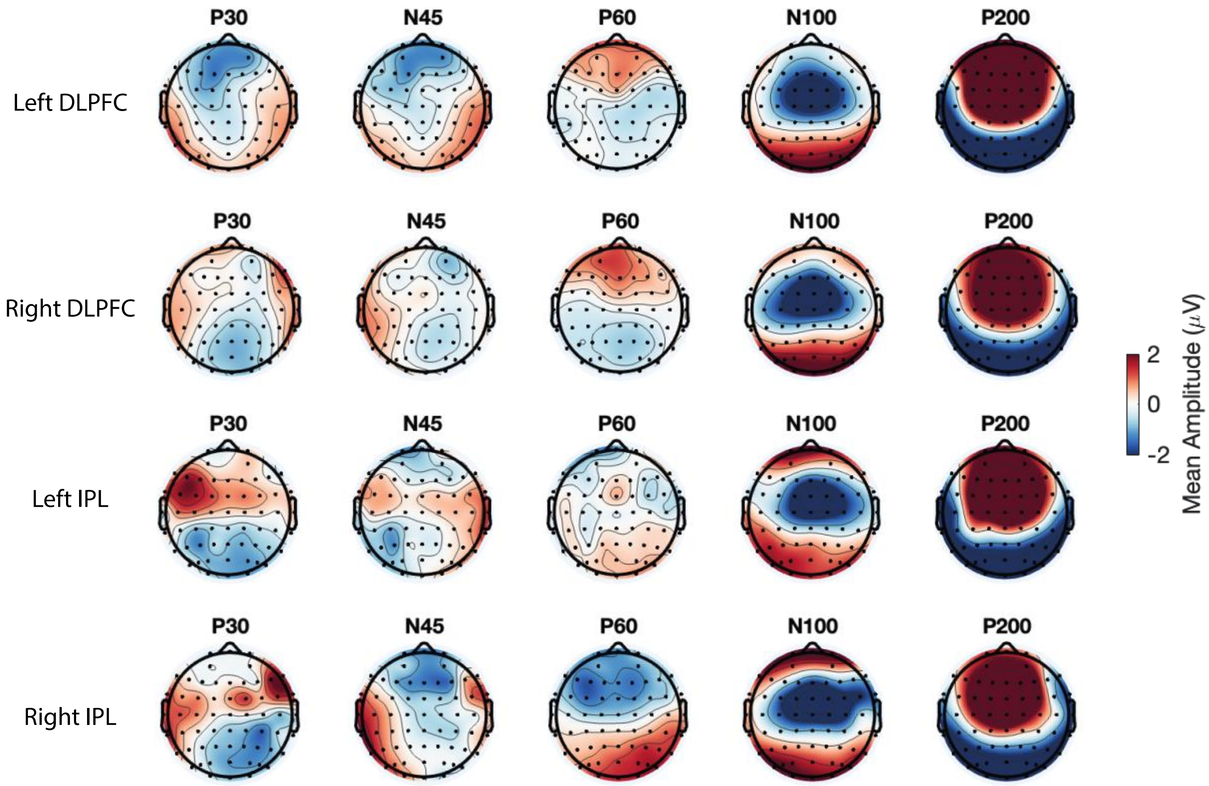

Supplementary Figure 5. Amplitude Topoplots Illustrate the TEPs as Obtained from Stimulation of the Four Cortical Cites in Clinical Trial One.

Supplementary Table 1. Fixed Effect Parameters as Obtained from the N45 Linear Mixed Effects Model for Electrode FC1 in Clinical Trial One.

Name	Estimate	Standard Error	t-Stat	Degrees of Freedom	p-Value	95% Confidence Intervals: Lower	95% Confidence Intervals: Upper
(Intercept)	14.0467	7.0617	1.9891	49	0.0523	-0.1444	28.2377
Age	0.3876	0.3302	1.1741	49	0.2460	-0.2758	1.0511
Sex (Female as Reference)	0.0584	1.6168	0.0361	49	0.9713	-3.1906	3.3074
Baseline N45	-0.1995	0.5675	-0.3515	49	0.7267	-1.3398	0.9409
Time (HRSD-17 Score After Treatment Session 5)	-3.9792	0.7908	-5.0316	49	< 0.0001	-5.5684	-2.3899
Time (HRSD-17 Score After Treatment Session 10)	-6.2441	0.8723	-7.1583	49	< 0.0001	-7.9971	-4.4912
Baseline N45: Time (HRSD-17 Score After Treatment Session 5)	1.6571	0.4357	3.8032	49	0.0004	0.7815	2.5326
Baseline N45: Time (HRSD-17 Score After Treatment Session 10)	2.0779	0.4636	4.4821	49	< 0.0001	1.1462	3.0095

Supplementary Table 2. Fixed Effect Parameters as Obtained from the P60 Linear Mixed Effects Model for Electrode FC1 in Clinical Trial One.

Name	Estimate	Standard Error	t-Stat	Degrees of Freedom	p-Value	95% Confidence Intervals: Lower	95% Confidence Intervals: Upper
(Intercept)	14.4046	6.3602	2.2648	49	0.0280	1.6232	27.1860
Age	0.3622	0.3035	1.1936	49	0.2384	-0.2476	0.9720
Sex (Male with Female as Reference)	0.5643	1.5296	0.3689	49	0.7138	-2.5095	3.6381
Baseline P60	0.2655	0.4291	0.6187	49	0.5390	-0.5968	1.1279
Time (HRSD-17 Score After Treatment Session 5)	-5.5682	0.7729	-7.2040	49	< 0.0001	-7.1215	-4.0149
Time (HRSD-17 Score After Treatment Session 10)	-8.1371	0.8144	-9.9912	49	< 0.0001	-9.7737	-6.5005
Baseline P60: Time (HRSD-17 Score After Treatment Session 5)	1.1245	0.3668	3.0652	49	0.0035	0.3873	1.8617
Baseline P60: Time (HRSD-17 Score After Treatment Session 10)	1.5423	0.4349	3.5467	49	0.0009	0.6684	2.4162

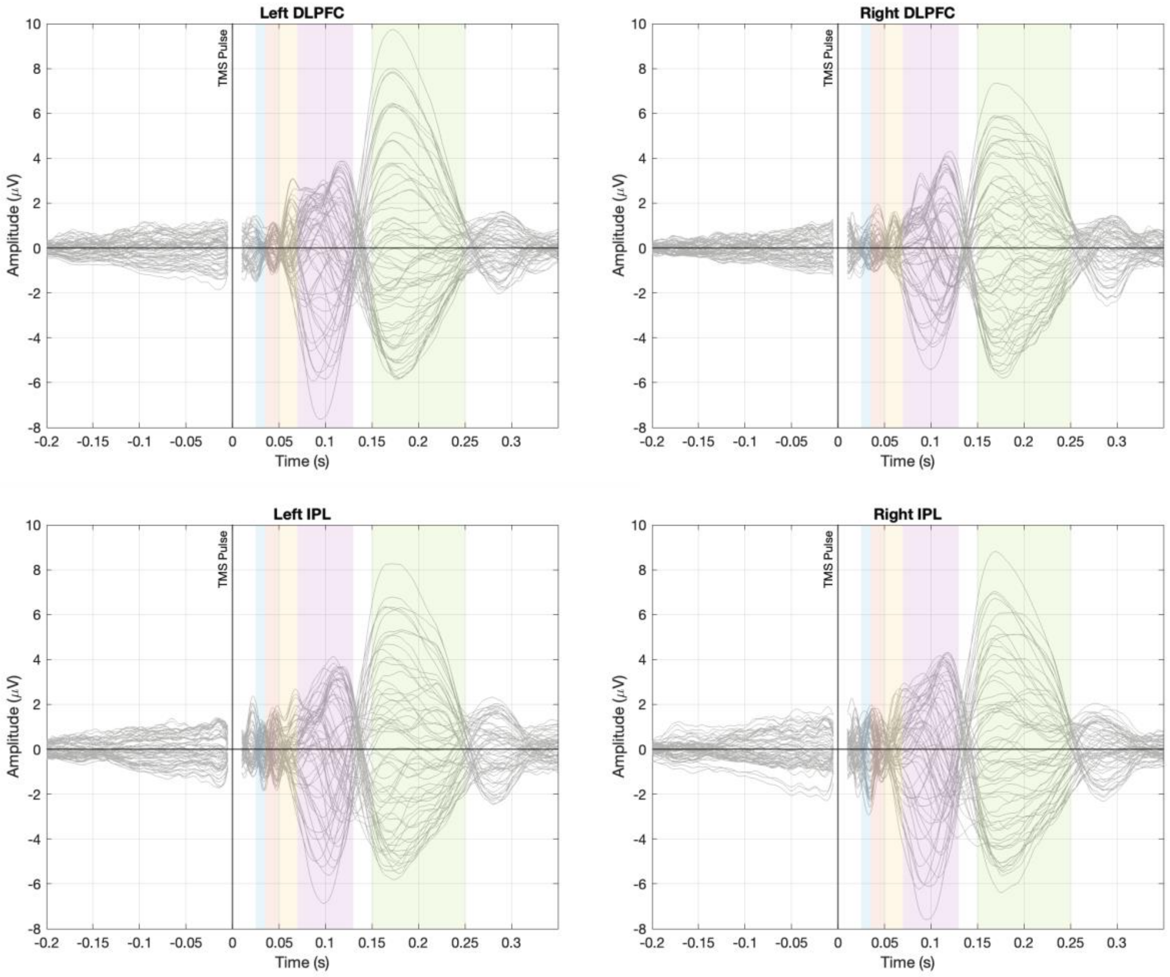

Supplementary Figure 6. TEP Plots Following Stimulation of the Four Cortical Sites in Clinical Trial Two. Butterfly plots illustrate the TEPs (averaged across all trials for each participant and then averaged across all participants) from all four cortical sites of interest, as obtained from clinical trial two. Each line represents an electrode. Different coloured shading represents the time windows used for calculating the mean amplitude for the P30, N45, P60, N100, and P200 TEPs.

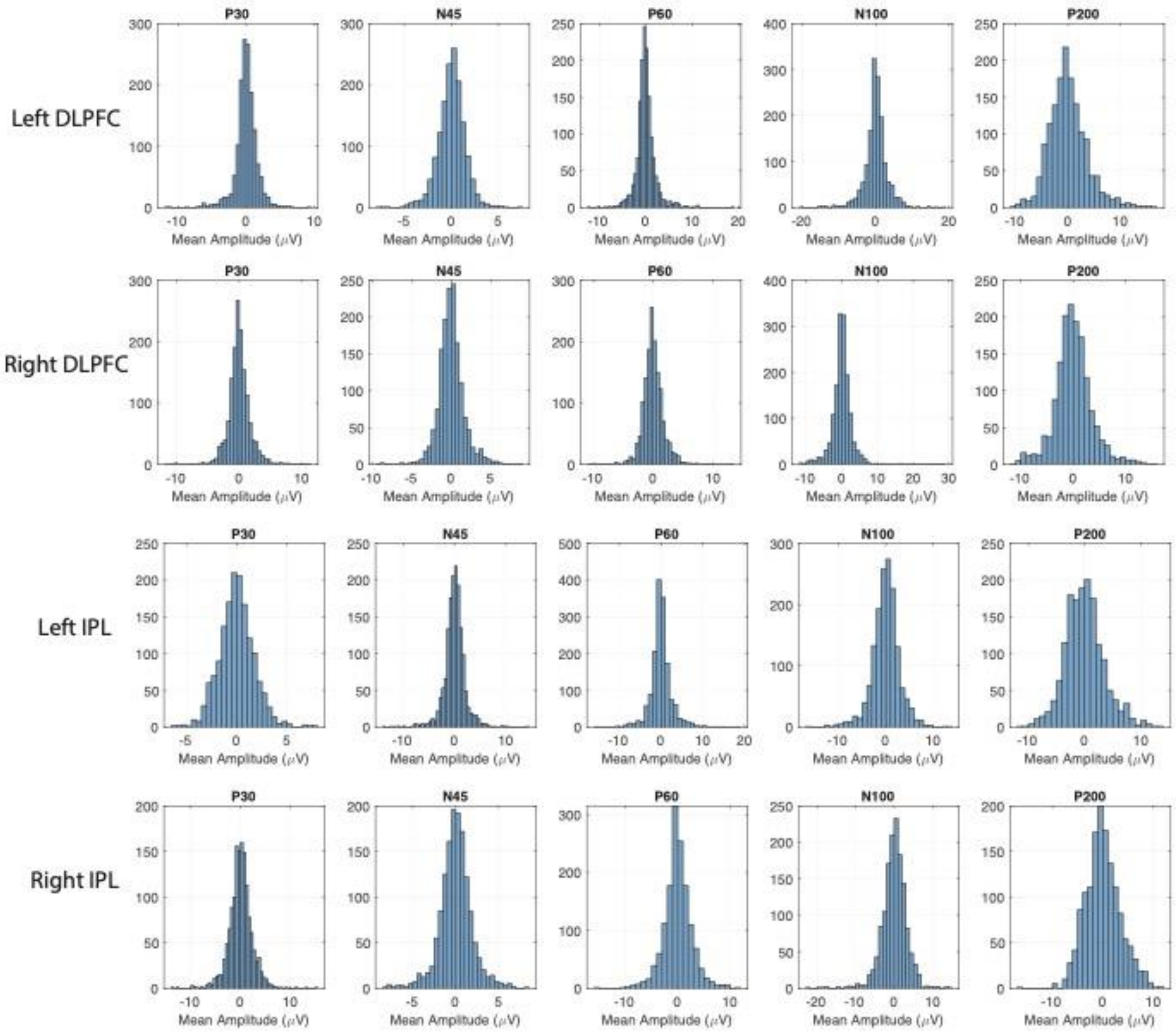

Supplementary Figure 7. Histogram Plots of all TEP Amplitude Values Following Stimulation of the Four Cortical Sites in Clinical Trial Two. Each histogram plot includes the TEP amplitude value from each electrode for each participant.

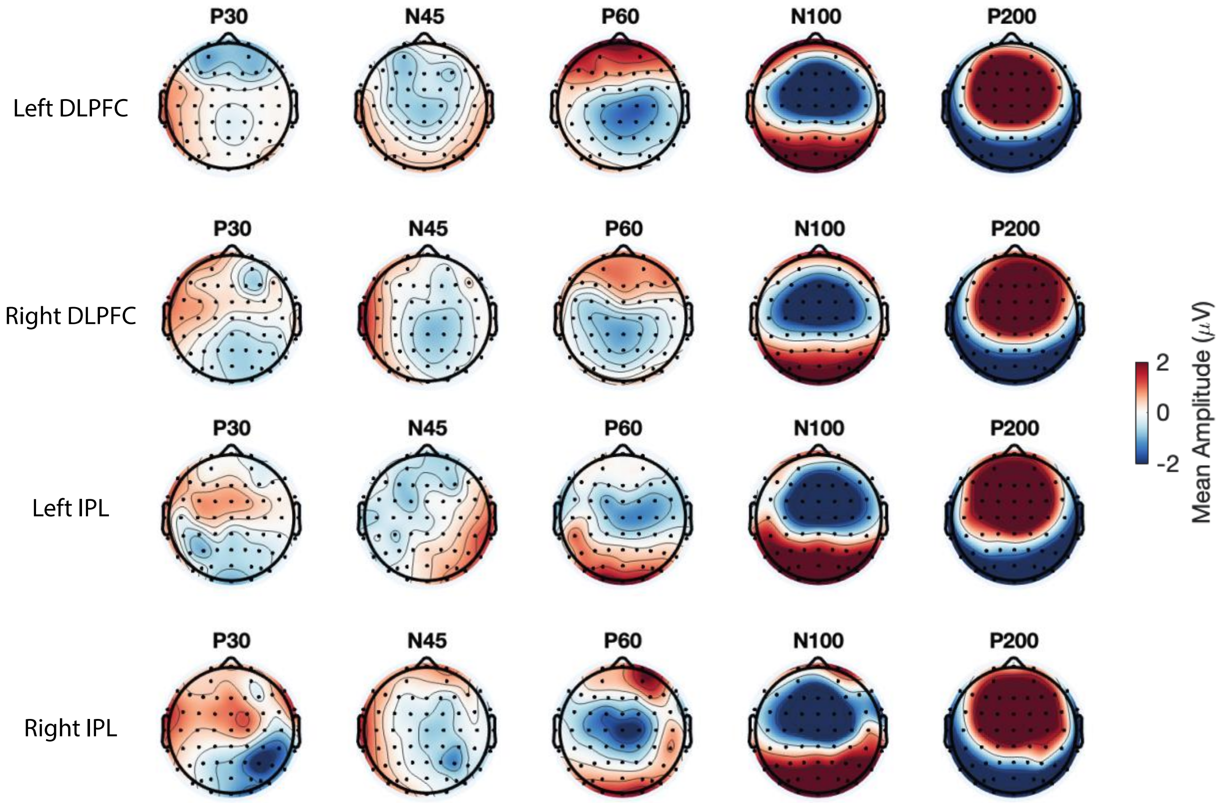

Supplementary Figure 8. Amplitude Topoplots Illustrate the TEPs as Obtained from Stimulation of the Four Cortical Cites in Clinical Trial Two.

Supplementary Table 3. Fixed Effect Parameters as Obtained from the N45 Linear Mixed Effects Model for Electrode FC3 in Clinical Trial Two.

Name	Estimate	Standard Error	t-Stat	Degrees of Freedom	p-Value	95% Confidence Intervals: Lower	95% Confidence Intervals: Upper
(Intercept)	14.6302	3.1563	4.6353	84	< 0.0001	8.3536	20.9069
Age	0.3403	0.1544	2.2048	84	0.0302	0.0334	0.6473
Sex (Males with Female as Reference)	0.0421	0.8185	0.0514	84	0.9591	-1.5856	1.6698
Sex (Transgender with Female as Reference)	1.8792	1.9619	0.9578	84	0.3409	-2.0223	5.7808
Baseline N45	-0.0898	0.4736	-0.1896	84	0.8501	-1.0316	0.8520
Time (HRSD-17 Score After Treatment Session 5)	-6.4932	0.8097	-8.0195	84	< 0.0001	-8.1033	-4.8830
Time (HRSD-17 Score After Treatment Session 15)	-9.1901	0.8324	-11.0407	84	< 0.0001	-10.8454	-7.5348
Time (HRSD-17 Score After Treatment Session 20)	-11.7942	0.8440	-13.9733	84	< 0.0001	-13.4726	-10.1157
Baseline N45: Time (HRSD-17 Score After Treatment Session 5)	-0.5860	0.5934	-0.9875	84	0.3262	-1.7660	0.5940
Baseline N45: Time (HRSD-17 Score After Treatment Session 15)	0.0800	0.6107	0.1310	84	0.8961	-1.1344	1.2944
Baseline N45: Time (HRSD-17 Score After Treatment Session 20)	2.0167	0.6111	3.2999	84	0.0014	0.8014	3.2320

Supplementary Table 4. Fixed Effect Parameters as Obtained from the P60 Linear Mixed Effects Model for Electrode F1 in Clinical Trial Two.

Name	Estimate	Standard Error	t-Stat	Degrees of Freedom	p-Value	95% Confidence Intervals: Lower	95% Confidence Intervals: Upper
(Intercept)	17.2164	2.9935	5.7512	84	< 0.0001	11.2635	23.1693
Age	0.2124	0.1472	1.4433	84	0.1526	-0.0803	0.5051
Sex (Males with Female as Reference)	0.0696	0.7490	0.0929	84	0.9262	-1.4199	1.5591
Sex (Transgender with Female as Reference)	2.5775	1.8242	1.4130	84	0.1614	-1.0501	6.2052
Baseline P60	0.0052	0.2613	0.0201	84	0.9840	-0.5144	0.5249
Time (HRSD-17 Score After Treatment Session 5)	-6.3259	0.8537	-7.4096	84	< 0.0001	-8.0237	-4.6282
Time (HRSD-17 Score After Treatment Session 15)	-10.0102	0.8803	-11.3710	84	< 0.0001	-11.7608	-8.2595
Time (HRSD-17 Score After Treatment Session 20)	-13.3170	0.8910	-14.9457	84	< 0.0001	-15.0889	-11.5451
Baseline P60: Time (HRSD-17 Score After Treatment Session 5)	0.1624	0.3439	0.4723	84	0.6379	-0.5215	0.8463
Baseline P60: Time (HRSD-17 Score After Treatment Session 15)	0.8567	0.3464	2.4728	84	0.0154	0.1677	1.5456
Baseline P60: Time (HRSD-17 Score After Treatment Session 20)	0.5121	0.3465	1.4781	84	0.1431	-0.1769	1.2010
